# Partial human Janus kinase 1 deficiency predominantly impairs responses to interferon gamma and intracellular control of mycobacteria

**DOI:** 10.3389/fimmu.2022.888427

**Published:** 2022-09-09

**Authors:** Vanessa Daza-Cajigal, Adriana S. Albuquerque, Dan F. Young, Michael J. Ciancanelli, Dale Moulding, Ivan Angulo, Valentine Jeanne-Julien, Jérémie Rosain, Ekaterina Minskaia, Jean-Laurent Casanova, Stéphanie Boisson-Dupuis, Jacinta Bustamante, Richard E. Randall, Timothy D. McHugh, Adrian J. Thrasher, Siobhan O. Burns

**Affiliations:** ^1^ Institute of Immunity and Transplantation, University College London, London, United Kingdom; ^2^ Department of Immunology, Royal Free London National Health Service (NHS) Foundation Trust, London, United Kingdom; ^3^ School of Medicine, Universidad Complutense, Madrid, Spain; ^4^ Department of Immunology, Hospital Universitario Son Espases, Palma, Spain; ^5^ Research Unit, Institut d’Investigació Sanitària de les Illes Balears (IdISBa), Palma, Spain; ^6^ School of Biology, University of St. Andrews, St. Andrews, United Kingdom; ^7^ St. Giles Laboratory of Human Genetics of Infectious Diseases, Rockefeller Branch, Rockefeller University, New York, NY, United States; ^8^ Molecular and Cellular Immunology Section, University College London Institute of Child Health, London, United Kingdom; ^9^ Department of Medicine, University of Cambridge, Cambridge, United Kingdom; ^10^ Laboratory of Human Genetics of Infectious Diseases, Necker Branch, National Institute of Health and Medical Research (INSERM) U1163, Paris, France; ^11^ Paris Cité University, Imagine Institute, Paris, France; ^12^ Howard Hughes Medical Institute, New York, NY, United States; ^13^ Study Center of Immunodeficiencies, Necker Hospital for Sick Children, Paris, France; ^14^ Research Department of Infection, University College London Centre for Clinical Microbiology, London, United Kingdom; ^15^ Immunology Department, Great Ormond Street Hospital for Children NHS Foundation Trust, London, United Kingdom

**Keywords:** JAK1, IFN immunity, immunodeficiency, mycobacterial disease, viral susceptibility

## Abstract

**Purpose:**

Janus kinase-1 (JAK1) tyrosine kinase mediates signaling from multiple cytokine receptors, including interferon alpha/beta and gamma (IFN-α/β and IFN-γ), which are important for viral and mycobacterial protection respectively. We previously reported autosomal recessive (AR) hypomorphic *JAK1* mutations in a patient with recurrent atypical mycobacterial infections and relatively minor viral infections. This study tests the impact of partial JAK1 deficiency on cellular responses to IFNs and pathogen control.

**Methods:**

We investigated the role of partial JAK1 deficiency using patient cells and cell models generated with lentiviral vectors expressing shRNA.

**Results:**

Partial JAK1 deficiency impairs IFN-γ-dependent responses in multiple cell types including THP-1 macrophages, Epstein-Barr Virus (EBV)-transformed B cells and primary dermal fibroblasts. In THP-1 myeloid cells, partial JAK1 deficiency reduced phagosome acidification and apoptosis and resulted in defective control of mycobacterial infection with enhanced intracellular survival. Although both EBV-B cells and primary dermal fibroblasts with partial JAK1 deficiency demonstrate reduced IFN-α responses, control of viral infection was impaired only in patient EBV-B cells and surprisingly intact in patient primary dermal fibroblasts.

**Conclusion:**

Our data suggests that partial JAK1 deficiency predominantly affects susceptibility to mycobacterial infection through impact on the IFN-γ responsive pathway in myeloid cells. Susceptibility to viral infections as a result of reduced IFN-α responses is variable depending on cell type. Description of additional patients with inherited JAK1 deficiency will further clarify the spectrum of bacterial and viral susceptibility in this condition. Our results have broader relevance for anticipating infectious complications from the increasing use of selective JAK1 inhibitors.

## Introduction

The widely expressed Janus kinase (JAK) family of tyrosine kinases are essential for signal transduction through interleukin (IL) and interferon (IFN) cytokine receptors. JAK family members (JAK1, JAK2, JAK3 and TYK2) associate with receptors individually or in pairs resulting in recruitment and phosphorylation of signal transducers and activators of transcription (STAT) proteins and transcription of STAT-responsive genes (1–3). In total the JAK/STAT pathway regulates multiple cellular functions including growth, differentiation and homeostasis although individual JAK/STAT molecules play specific roles in different cell types (4).

The roles of several members of the JAK family for immune cell function have been clarified through investigation of human and murine deficiency states (5, 6). For example, autosomal recessive (AR) complete JAK3 deficiency typically cause severe combined immunodeficiency (SCID) characterized by absence of autologous T and NK cells, highlighting the importance of JAK3 signaling from interleukin (IL)-2 receptor subunit gamma (IL-2RG)-containing IL receptors for development of these lineages (7–10). On the other hand, AR complete TYK2 deficiency result in susceptibility to mycobacterial and viral disease as a result of impaired signaling from the IL-12/IL-23/IL-10 and IFN-α/β receptors respectively (5, 11–13). AR partial P1104A-TYK2 deficiency has limited predisposition to mycobacterial disease due to selectively impaired responses to IL-23 but not other cytokines (13, 14), highlighting that complete and partial deficiencies can have different cellular and clinical phenotypes. Autosomal dominant (AD) JAK1 gain of function (GOF) mutation was reported in patients with severe multisystem autoinflammatory disease (15). However, defining the specific effect of JAK1 deficiency on the immune system has been hampered by perinatal lethality in murine models of complete JAK1-deficiency as a result of neurological defects (1).

We have reported the first, and to date only, case of human inherited AR partial JAK1 deficiency associated with two germline homozygous missense mutations in the pseudokinase domain of JAK1 (16). Together these mutations had a hypomorphic effect on JAK/STAT signaling associated with reduced kinase function and a slight reduction in JAK1 protein expression (16, 17). Although JAK1 cooperates with JAK3 for common gamma chain (γc) receptor signaling, lymphocyte development and function were relatively well preserved. Instead, recurrent atypical mycobacterial disease was the dominant clinical phenotype, grouping JAK1 deficiency with other diverse genetic defects of the IFN-γ immunity as a cause of syndromic mendelian susceptibility to mycobacterial disease (MSMD, **Supplementary Table 1**), (18). Somewhat surprisingly given the partnership of JAK1 with TYK2 for IFN-α/βR signaling (12, 19–26), serious viral infections were not seen. Flat forehead warts, which are unusual in immunocompetent individuals, were present but not sampled for virus identification. These features suggest a non-redundant role for JAK1 in IFN-γR signaling but its relative importance for IFN-α/βR signaling remains unclear.

In our previous analysis of AR partial JAK1 deficiency, responses to IFN-α and IFN-γ were defective in primary dermal fibroblasts and in whole blood analysis (16). As partial deficiency of another JAK family protein, TYK2, has different impact depending on the specific cell type examined (13), we set out to expand our previous findings and test the impact of partial JAK1 deficiency on pathogen control. Here, using a combination of patient cells and knock down cell lines, including fibroblasts, B- and myeloid cells, we specifically investigate the impact of partial JAK1-deficiency on IFN signaling in hematopoietic and non-hematopoietic cell lineages.

## Materials and methods

### Patients cells and human cell lines

THP-1 cells were obtained from the American Type Culture Collection (ATCC #TIB-202). EBV-B cells from patients and healthy controls were derived from peripheral blood mononuclear cells (PBMCs) (27). Informed written consent was obtained in accordance with the Declaration of Helsinki and ethical approval from the Great Ormond Street Hospital for Children NHS Foundation Trust and the Institute of Child Health Research Ethics Committee (Reference Number: 06/Q0508/16). For cell culture details, see Supplemental Methods.

### Lentivirus preparation and transductions

JAK1 knock down (KD) and scrambled control (Sc) cell lines were generated using lentiviral vectors expressing short hairpin RNA (shRNA) sequences as previously described (17). For further information, see Supplemental Methods.

### Determination of mRNA levels by real time-quantitative polymerase chain reaction and reverse transcription polymerase chain reaction

THP-1 cells were stimulated or left unstimulated with 50 ng/ml IFN-γ (Invitrogen) for 24h. Total RNA from cells was extracted using RNAeasy kit (Qiagen) and converted to cDNA by reverse-transcription using Quantitect reverse transcription kit (Qiagen). Determination of mRNA level was performed by RT-PCR using specific primers (**Supplementary Table 2**) and QuantiTect SYBR^®^ Green PCR Kit (Qiagen) according to manufacturer’s instructions. Fold changes were calculated using the DDCT2 (-Delta Delta C(T)) method and results normalized with respect to the values obtained for the endogenous *ACTIN* and *GAPDH* cDNA. See supplemental methods for assessment of *MX1* and *OAS1* following IFN-α stimulation.

### Flow cytometry analysis of STAT phosphorylation

THP-1 cells were stimulated with 10^3^ IU/ml IFN-α2b or 50 ng/ml IFN-γ. EBV- B cells were stimulated with 10^5^ IU/ml IFN-α2b or 500 ng/ml IFN-γ for 10 min. Cells were fixed using fix buffer I and permeabilized using Perm Buffer III (BD Biosciences) for 30 min at 4°C, and labelled with anti-pSTAT1 (clone 4a, BD Biosciences) or anti-STAT1 (clone 1/Stat1, BD Biosciences) antibodies for 60 min at room temperature. At least 10000 gated events were acquired on a BD LSRFortessa cytometer and data were analyzed using FlowJo software (Tree Star Inc., USA). Data on graphs is shown as relative increase (mean fluorescence intensity (MFI) of stimulated cells – MFI of unstimulated cells/MFI unstimulated cells).

### Infection models with bacteria *in vitro*

For details of *Mycobacterium bovis Calmette–Guérin* (BCG) and *Salmonella typhimurium*, including culture, see Supplemental Methods. THP-1 cells were differentiated into macrophages using 10 ng/ml of phorbol myristate acetate (PMA) for 48 h and then were left unstimulated or stimulated with IFN-γ 50 ng/ml for 18 h before infection. Cells were infected using stocks (BCG) or bacteria in mid-log grow phase (*Salmonella*), using a multiplicity of infection (MOI) of 20:1 for BCG expressing-mCherry and 10:1 for BCG and *Salmonella*. Monolayers were incubated for 4 h with BCG and 30 min with Salmonella at 37°C in 0.5% CO_2_. Infected cells were washed to remove extracellular bacteria. To kill extracellular bacteria after *Salmonella* infection, cells were incubated in complete medium with Gentamicin (100 µg/ml) for two hours. Subsequently, macrophages were incubated in fresh complete medium, in the presence or absence of IFN-γ (50 ng/ml) for different time points (detailed in the legends).

### Harvest of infected macrophage lysate for CFU plating

Cells were lysed at 3 days for BCG infection or 24 h for *Salmonella* infection with 0.05% SDS w/v in H_2_O and serial dilutions plated out on Middlebrook 7H11 or LB agar plates followed by incubation at 37°C for 14 days for BCG infection or 12 h at 37°C after *Salmonella* infection. Bacterial survival, measured as CFU for each condition, was expressed as a percentage of the CFU counted in the untreated Sc control.

### Quantification of the infected cells by flow cytometry

Macrophages differentiated from Sc and KD THP-1 cell lines using PMA, were left unstimulated or stimulated with IFN-γ (50ng/ml) before infection with BCG expressing-mCherry strains (MOI 20:1). After phagocytosis, cells were washed and incubated in complete medium in the presence or absence of IFN-γ (50 ng/ml) for the given time points. Cells were removed from the plate using Accutase^®^ solution (A6964, Sigma Aldrich), washed with PBS, fixed in 4% paraformaldehyde (PFA) for 10 min and analyzed by flow cytometry (BD LSRFortessa) using FlowJo.

### Microscopy

200,000 THP-1 cells were differentiated on 35mm glass bottom dishes (Fluorodish) for microscopy experiments. For further details see Supplemental Methods.

### pH sensitivity of pHrodo-labelled BCG

BCG-lux were labelled with pHrodo™ (Invitrogen) at a concentration of 25mM according to the manufacturer’s instructions, except for omission of the 100% methanol step. Approximately 100,000 CFU were resuspended in 500 µl buffer at pH 7. Samples were then acquired on a BD Fortessa flow cytometer (BD LSRFortessa), and pHrodo fluorescence was measured in the PE-Texas Red channel, and analyzed using FlowJo.

### Apoptosis assays

Macrophages were left unstimulated or stimulated with IFN-γ (50ng/ml) before BCG infection (MOI 10:1). Percentage of apoptosis was determined using APC Annexin V apoptosis detection kit with PI (BioLegend 640932) according to manufacturer’s instructions by flow cytometry (BD LSRFortessa), and analyzed using FlowJo.

### Viral assays

Primary dermal fibroblast and EBV-B cells viral assays were performed as previously described (24, 28–31). For further details see Supplemental Methods.

### Statistical analysis

Statistical analysis was performed with Graph Prism Version 5.01. The following tests were used: One-way ANOVA; Wilcoxon-Signed Rank for pairwise comparisons; Mann-Whitney for unpaired comparisons. Results were expressed as mean ± SEM. *P*-values < 0.05 were considered significant.

## Results

### Partial JAK1 deficiency impairs STAT1 phosphorylation and expression of IFN-γ-inducible genes in THP-1 cells

**Figure 1:**
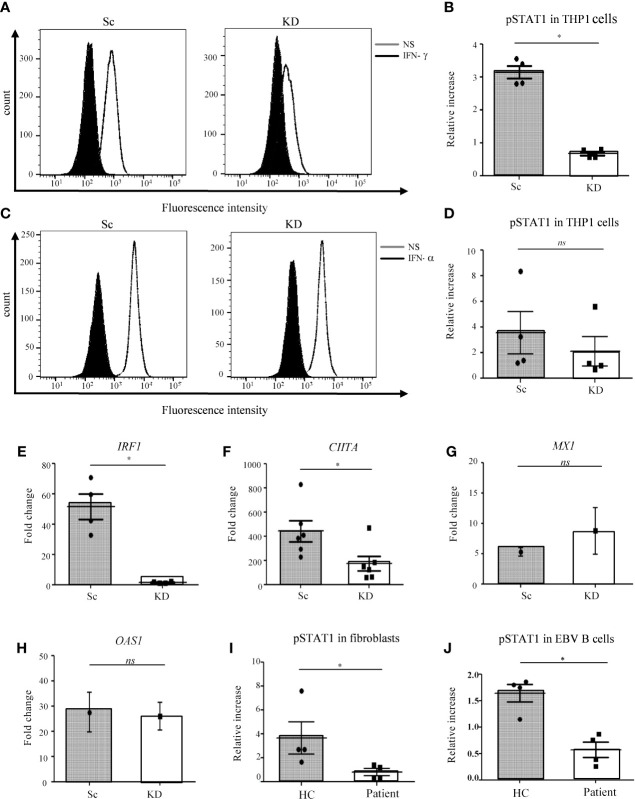
STAT1 responses are impaired in JAK1-deficient THP-1 cells, fibroblasts and EBV-B cells. **(A–D)** Analysis of JAK/STAT signaling by flow cytometry (FC) in THP-1 cells after IFN-γ/IFN-α stimulation. **(A)** and **(C)** display a representative experiment, **(B)** and **(D)** are from four independent experiments. Two-tailed Mann Whitney test. **(E, F)** RTqPCR analysis of *IRF1* and *CIITA* expression from THP-1 cells after IFN-γ stimulation. Data is from five independent experiments. Two-tailed Mann Whitney test. **(G, H)** RT-qPCR analysis of *MX1* and *OAS1* expression in THP-1 cells after stimulation with 1000 IU/ml IFN-α. Graphs represent the mean of three experiments. Data was compared using unpaired t-test. **(I, J)** Analysis of JAK/STAT signaling by FC in control and patient fibroblasts and EBV-B cells after stimulation with IFN**-γ**. Data are from four independent experiments. Two-tailed Mann Whitney test. *P <0.05; NS, not significant.

As the patient with AR partial JAK1 deficiency presented predominantly with MSMD (**Table S1**), we sought to establish a model to examine the role of JAK1 in myeloid cells during mycobacterial infection. As patient blood was not accessible, for this study we generated a THP-1 monocytic cell line with sub-total JAK1 knock-down (KD) to model partial JAK1 deficiency using lentiviral vectors expressing short hairpin RNA (shRNA) sequences. The JAK1 KD model does not fully recapitulate the impact of the patient’s variants which preserved expression of mutant JAK protein that had reduced signaling function (16) but allows assessment of reduced JAK1 function by reducing protein expression. Compared to control shRNA, *JAK1* shRNA substantially reduced *JAK1* messenger RNA expression for three out of four hairpins tested (**Figure S1A**). THP-1 cells transduced with *JAK1* shRNA #3 were utilized for further studies. Compared with shRNA control cells, 25-30% JAK1 protein expression was detected in *JAK1* shRNA #3 cell lines using western blotting, in keeping with partial knock down (**Figure S1B**). To test whether the level of JAK1 reduction was sufficient to impair JAK1 protein function, we studied JAK1-mediated phosphorylation of STAT1 in response to IFN-γ stimulation, using flow cytometry. We observed a significant decrease in STAT1 phosphorylation following IFN-γ stimulation in the THP-1 JAK1-KD cells compared to scrambled control shRNA cell lines (Sc) (p<0.05) (**Figures 1A, B**). Surprisingly, STAT1 phosphorylation in response to IFN-α stimulation was not significantly reduced (**Figures 1C, D**), suggesting that partial impairment of JAK1 function affects predominantly the IFN-γ response in THP-1 cells. Following stimulation with IFN-γ, upregulation of interferon regulatory factor 1 (*IRF1*) and Class II Transactivator (*CIITA*) mRNA was significantly lower in the KD than Sc lines, indicating impaired downstream gene transcription in JAK1 deficiency (**Figures 1E, F**). In contrast, upregulation of 2’-5’-oligoadenylate synthetase 1 (*OAS1*) and *MX1* in response to IFN-α stimulation was preserved (**Figures 1G, H**). Our findings were unlikely to be due to alterations in the surface expression of IFN-γ or IFN-α receptors as surface levels of IFNGR1 and IFNAR2 were comparable between JAK1 KD and Sc cell lines at baseline and after stimulation with their ligand (**Figures S2A–D**). As seen in THP-1 cells, partial JAK1 deficiency had a clear impact on IFN-γ signaling in primary dermal fibroblasts and EBV B-cells from the patient with AR JAK-1 deficiency, resulting in significantly reduced STAT1 phosphorylation following IFN-γ stimulation (**Figures 1I, J**) and (**Figures S3A, B**), albeit to a lesser degree that seen in fibroblasts from a patient with complete IFN-γR deficiency (16) (**Figure S3B**).

### Partial loss of JAK1 function enhances mycobacterial and salmonella survival in myeloid cells

**Figure 2:**
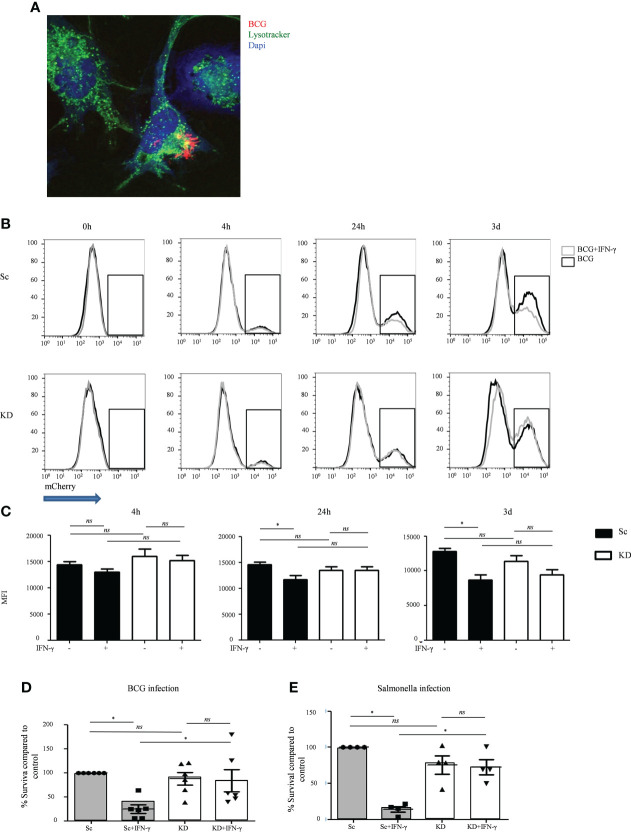
JAK1-deficient THP-1 cells show increased mycobacterial and *Salmonella* survival after IFNγ stimulation**. (A)** Internalization of mCherry-BCG by THP-1 cells, unstimulated or stimulated with IFN-γ. A is from a representative experiment**. (B, C)** FC quantitation of mCherry-BCG in THP-1 cells; mCherry fluorescence was measured in the PE-Texas Red channel. Black line – BCG infected cells, gray line – BCG infected cells + IFN-γ stimulation. B displays a representative experiment; C is from five independent experiments. **(D, E)** Bacterial survival in THP-1 cells infected with BCG or *Salmonell*a strains, with or without IFNγ stimulation. Data is from six and four independent experiments respectively. Two-tailed Mann Whitney test. *P <0.05; NS, not significant.

To test the impact of reduced JAK1 function on IFN-γ-mediated host defense to intracellular pathogens we utilized BCG as a well-established model for mycobacterial infection (32). Macrophages differentiated from the THP-1-JAK1 KD and Sc cell lines were infected with BCG, with or without prior IFN-γ stimulation. JAK1 KD and Sc THP-1 cells were capable of internalizing BCG, as seen by confocal microscopy (**Figure 2A**). Using confocal analysis and a lysotracker dye which increases fluorescent intensity in low pH (33), both Sc and JAK1 KD cell lines were observed to traffic a proportion of internalized BCG into acidified compartments (**Figure S4A**).

To better quantitate BCG infection, THP-1 macrophages were co-cultured with mCherry-expressing BCG and analyzed by flow cytometry. Similar levels of bacteria were internalized by KD and Sc lines at 4 hours, and this was largely unaffected by IFN-γ stimulation (**Figures 2B, C**), indicating that loss of JAK1 does not significantly impact phagocytosis. As expected, at both 24 and 72 hours, IFN-γ stimulation significantly reduced mCherry fluorescence in Sc lines consistent with lower bacterial survival. In contrast, IFN-γ had no significant impact on mCherry levels in KD lines (**Figures 2B, C**). To confirm that JAK1 deficiency promotes intracellular BCG survival, KD and Sc lines were lysed on culture plates 3 days after infection and BCG titers were quantitated by counting colony forming units (CFU/ml). As seen in flow cytometry assays, BCG survival was higher in KD lines indicating an important role for JAK1 in controlling mycobacterial infection (**Figure 2D**). Similar findings were obtained with *Salmonella typhimurium* (**Figure 2E**), another intracellular pathogen known to require IFN-γ for control of the bacterial infection, with significantly higher bacterial survival seen in KD than Sc cells. Together these results demonstrate that JAK1-deficient myeloid cells permit enhanced intracellular mycobacterial and salmonella survival *in vitro*.

### Partial JAK1 deficiency impairs IFN-γ-induced phagosome acidification and apoptosis in myeloid cells

**Figure 3:**
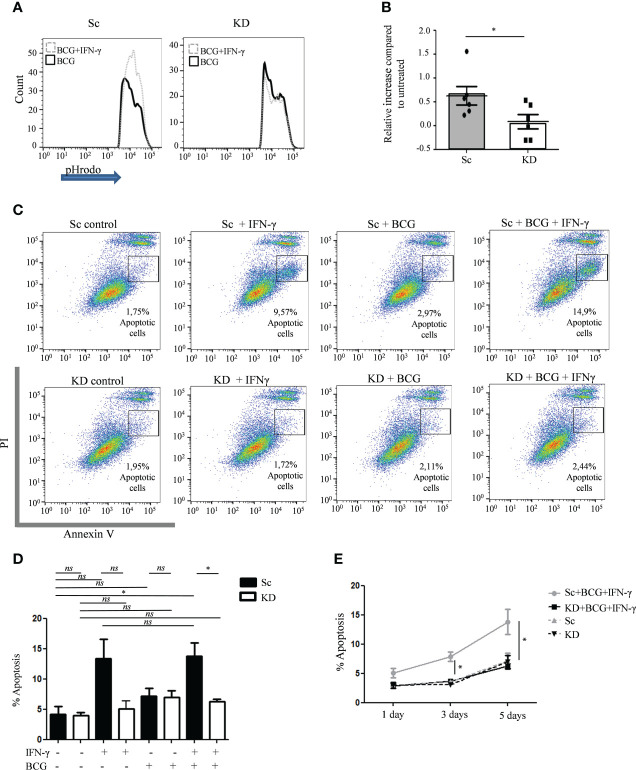
Phagosome acidification and apoptosis is reduced in JAK1-deficient THP-1 cells. **(A, B)** FC measurement of phagosome acidification using detection of pHrodo-labelled BCG post infection of THP-1 cells, with or without prior IFN-γ stimulation. Data is from six independent experiments. **(C–E)** Percentage of apoptosis quantified by FC using annexin V/PI staining in THP-1 cells at different time points following BCG infection, with or without IFN-γ stimulation. C displays a representative experiment 5 days post infection. D and E are from four independent experiments, showing 5 day **(D)** and 1, 3 and 5 day **(E)** timepoints. Statistical comparisons in E are for Sc+BCG+IFN *vs*. KD+BCG+IFN. Two-tailed Mann Whitney test. *P <0.05; NS, not significant.

To further explore the mechanisms promoting enhanced bacterial intracellular survival in myeloid cells with reduced JAK1 function, phagosome acidification and apoptosis were tested as these are key IFN-γ-dependent steps in the control of mycobacterial infection (34–39). Following infection of THP-1-derived macrophages with pHrodo-labelled BCG, phagosomal acidification was measured by measuring fluorescence, which is released in the context of low pH. Even in the absence of IFN-γ stimulation, both Sc and KD THP-1 cells had relatively high levels of pHrodo fluorescence, which relates to the high baseline lysosomal content in PMA differentiated THP-1 macrophages (40) (**Figure 3A** and **Figure S4B**). Fluorescence intensity was increased after IFN-γ-stimulation in Sc cells lines consistent with additional IFN-γ-mediated induction of acidification (**Figures 3A, B**). In contrast there was no increase in acidification in the KD cell line following IFN-γ stimulation.

To test whether partial JAK1 deficiency is sufficient to impair IFN-γ-induced apoptosis, Annexin V/PI staining was measured by flow cytometry. Sc control and KD THP-1 cells had similar baseline levels of apoptosis which was not significantly increased 5 days after BCG infection alone (**Figures 3C, D**). In contrast, IFN-γ pre-treatment induced significant apoptosis at both 3 and 5 days after BCG infection in Sc control cells compared with untreated Sc control cells, an effect that was abrogated in KD cells (**Figures 3C, E** and **Figure S5**). Together our data suggest that defective intracellular bacterial killing in myeloid cells with reduced JAK1 function is at least in part due to impaired IFN-γ-induced phagosome maturation and apoptosis.

### Partial JAK1 deficiency impairs anti-viral response in EBV-B cells but not in fibroblasts

**Figure 4:**
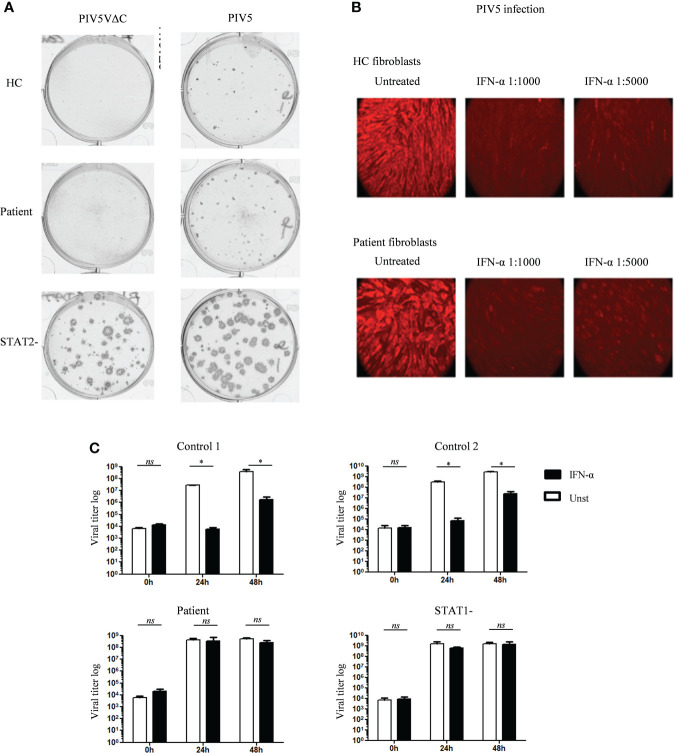
Variable *in vitro* antiviral response in fibroblasts and EBV B cells of the patient with JAK1 deficiency **(A)** Relative plaque sizes of the PIV5/PIV5VΔC virus visualized by immunostaining in fibroblasts from patients with partial JAK1, complete STAT2 deficiency and healthy control. **(B)** Visualization of PIV5 virus-infected cells by immunofluorescence in control and JAK1 deficient patient fibroblast, with or without IFN-α pre-treatment. Data display a representative experiment from three independent experiments. **(C)** Determination of VZV viral load in EBV-B cells from the patient with partial JAK1, complete STAT1 deficiency, and two healthy controls (C1 and C2), with or without pre-treatment with IFN-α. Data is from three independent experiments. One-tailed Mann Whitney test. *P <0.05; NS, not significant.

To test viral susceptibility in JAK1 deficient cells, we utilized established viral infection models in both fibroblast and EBV-B cells (24, 41, 42). We have previously demonstrated reduced STAT1 phosphorylation following IFN-α stimulation in primary dermal fibroblasts from the patient with AR partial JAK1 deficiency (16). We also observed reduced STAT1 phosphorylation following IFN-α in EBV-B cells from the same patient with AR partial JAK1 deficiency, albeit to a lesser degree than that seen in IFNAR-deficiency (**Figures S6A, B**). For fibroblast infections, we used Parinfluenza virus 5 (PIV5) and highly attenuated recombinant strains of PIV5 (PIV5VΔC) that lack defined functional IFN antagonists (33, 34). This virus is weakly virulent forming only pinpoint plaques in cells that produce and respond to IFN but with ability to form large plaques if the IFN system is impaired. As previously shown (41), fibroblast monolayers from patients with complete STAT2 deficiency supported the formation of large plaques (infected cells) of PIV5 and PIV5VΔC, demonstrating uncontrolled viral infection resulting from failure of the IFN-α response (**Figure 4A**). Fibroblast monolayers from heathy control and the patient with partial JAK1 deficiency prevented large viral plaque formation indicating successful viral control (**Figure 4A**). This assay relies on production of endogenous IFN-α by infected fibroblasts and therefore reflects cellular response to physiological concentrations of IFN.

We also tested whether partial JAK1 deficiency altered the capacity of fibroblasts to respond to exogenous addition of IFN-α to control PIV5 infection. Using immunostaining to visualize intracellular virus, loss of viral fluorescence was seen during successful suppression of viral infection in healthy control fibroblasts treated with IFN-α (**Figure 4B**). Comparable viral suppression was mediated by patient fibroblasts after IFN-α stimulation suggesting partly preserved IFN-α responses in fibroblasts with reduced JAK1-function (**Figure 4B**). Using a separate model, fibroblasts from healthy controls and patient were infected with herpes simplex virus-1 (HSV-1), showing control of viral infection 24h after treatment with exogenous IFN-α (**Figure S7**).

To further test the impact of partial JAK1 deficiency on host viral protection we used a separate model in which EBV-B cells are infected with VSV. As expected, healthy control EBV-B cells controlled viral infection when treated with exogenous IFN-α, evidenced by lower viral titers compared with untreated cells at 24h and 48h after infection (**Figure 4C**). In contrast, EBV-B cells from the patient with AR partial JAK1-deficiency exhibited no reduction in viral titers in the presence of IFN-α indicating a significantly reduced response to IFN-α. In this assay, VSV titers in infected patient EBV-B cells were comparable to STAT1-deficient EBV B-cells after 24h and 48h of infection (**Figure 4C**). Together these results suggest that partial JAK1 deficiency results in impaired viral protection with variable impact according to the cell type involved.

## Discussion

An increasing number of disease-causing mutations have been described in type I and type II IFN pathways (5, 18, 21, 25, 26, 41, 43–46) (**Table S1**). Defects of IFN-γ-mediated immunity give rise to mycobacterial susceptibility (MSMD) while loss of IFN-α/β function results in viral infection of varying severity, ranging from relatively mild to fatal disease (18, 21, 24, 25, 41, 42, 45–49). Typically, overlap of mycobacterial and viral phenotypes occurs where the defective signaling molecule is shared by both pathways, for example in TYK2 or STAT1 deficiency (24), although the degree of infection susceptibility depends on how and to what extent signaling is impaired (50). We previously identified homozygous mutations in *JAK1* causing AR partial JAK1 deficiency in a patient with mycobacterial susceptibility (16). Although JAK1 is shared by both IFN-γ and IFN-α/β/λ signaling pathways, severe viral infections were absent into adulthood. In contrast with other inherited defects of IFN-α/β/λ signaling (43), vaccine strain measles, mumps and rubella as well as wildtype CMV, EBV and VZV were tolerated normally in this patient without serious clinical disease.

Here we investigated the specific impact of partial JAK1-deficiency on IFN-γ and IFN-α/β signaling using several different cell line models generated either from our patient, where mutations in the pseudokinase domain resulted in a slightly reduced level of JAK1 protein which lacked full kinase function (16), or using shRNA to reduce wild type JAK1 protein expression. While the shRNA JAK1 KD model does not fully recapitulate the patient’s variants, it remains a useful model to assess the effects of partial JAK1 function on cell function. Using *in vitro* infection models with BCG and *Salmonella*, myeloid lineage THP-1 cells generated using shRNA to achieve partial JAK1-deficiency (25-30% residual JAK1 protein expression) supported enhanced bacterial survival after IFN-γ stimulation, reminiscent of uncontrolled mycobacterial replication previously reported in IFN-γR1-deficient human iPSC-derived macrophages (51). IFN-γ-activated healthy macrophages are more resistant to mycobacterial infection by the induction of several discreet mechanisms that promote mycobacterial killing (52–55), such as expression of IFN-γ-inducible genes (*IRF1* and *CIITA*), phagosome maturation and apoptosis (35–37, 56, 57), all of which were found to be reduced in the JAK1 knock down cell line after IFN-γ stimulation. Therefore, we concluded that, in myeloid cells, JAK1 is non-redundant for multiple aspects of the IFN-γ-response required to control intracellular bacterial infection. Our data shows that partial disruption of JAK1 signaling is sufficient to impair anti-mycobacterial protection, which has implications for the expected phenotype of hypomorphic JAK1 mutations and for the increasing use of JAK1 inhibitors in other areas of medicine. In our THP-1 macrophage model, expression of JAK1 protein was reduced by 70-75% which significantly reduced STAT1 phosphorylation and gene expression following IFN-γ stimulation but supported relatively normal pSTAT1 and gene expression after IFN-α stimulation. Further work is required to determine what levels of residual JAK1 expression and signaling function are required to preserve immune competence against mycobacteria *in vitro* and, more importantly, *in vivo*.

Given the known role of JAK1 in signaling from the IFN-α/β receptor, we studied the ability of dermal fibroblasts and EBV-B cells from the JAK1-deficient patient to develop antiviral responses *in vitro*. Surprisingly, despite a documented reduction in STAT1 signaling in patient primary dermal fibroblasts (16), we found no detectable susceptibility to viral infection using three different viruses, suggesting that residual JAK1 activity was sufficient to preserve sufficient IFN-α response for the control of viral proliferation in that cell type. In contrast, the patient’s EBV-B cells showed lack of viral protection following VSV infection, which may indicate a more pronounced impact of partial JAK1 deficiency on the type I IFN response in hematologic cells. Although in our patient we observed only flat warts (which were presumed to be due to HPV) without life-threatening viral infections, the description of additional patients may broaden the phenotype of JAK1 deficiency in humans and provide opportunities to further assess the relative importance of JAK1 for viral protection in hematopoietic and non-hematopoietic cell types. Future studies should also address the impact of JAK1 deficiency on IFN-λ signaling which is important for viral protection in epithelial cells, natural killer and dendritic cells (58). It remains possible that patients with JAK1 deficiency retain relatively normal viral susceptibility in specific cell types *in vivo* as result of other antiviral mechanism, as has been suggested for patients with complete STAT2 deficiency who also have surprisingly mild viral infections (41).

Here we provide the first evidence that partial loss of JAK1 function results in mycobacterial susceptibility by reducing multiple aspects of the IFN-γ response in myeloid lineage cells. Our data suggest that the predominant effect of partial JAK1 deficiency is on the IFN-γ pathway, as IFN-α but not IFN-γ responses were preserved in our shRNA model despite 70-75% loss of JAK1 expression. Although viral susceptibility was also observed *in vitro*, this varied according to cell type. Our findings contrast with the adverse effect profile published with early trails of the selective JAK1 inhibitors, filgotinib and upadacitinib, where herpes zoster viral infections and not mycobacterial disease predominate (59–62). However, even though filgotinib is considered a JAK1 selective inhibitor, it still has a role in inhibiting other JAKs with IC50 of 10 nM, 28 nM, 810 nM, and 116 nM for JAK1, JAK2, JAK3, TYK2, respectively. This possible inhibitory effect to the other JAKs may influence viral susceptibility (63). More extensive use of the JAK1/JAK2 inhibitor ruxolitinib is associated with a greater risk of mycobacterial infections (*Mycobacterium tuberculosis* and atypical mycobacterial infections) in the treatment of patients with myelofibrosis and polycythemia vera (64, 65).

We support a recommendation that previous mycobacterial infection should be investigated when considering the use of JAK inhibitors (66) and suggest tuberculin skin testing and an IFN-γ release assay (IGRA) prior to the prescription of JAK1 inhibitors. Longer experience with pharmacological JAK1 inhibition and identification of additional patients with germline JAK1 deficiency, including perhaps patients with more common and milder forms of JAK1 deficiency as recently shown for TYK2 (13), will allow us to better understand the relative importance of JAK1 for specific cytokine pathways governing host protection *in vivo*.

## Data availability statement

The original contributions presented in the study are included in the article/**Supplementary Material**. Further inquiries can be directed to the corresponding author.

## Ethics statement

Ethical approval was obtained from the Great Ormond Street Hospital for Children NHS Foundation Trust and the Institute of Child Health Research Ethics Committee (Reference Number: 06/Q0508/16). The patients/participants provided their written informed consent to participate in this study. Written informed consent was obtained from the individual(s) for the publication of any potentially identifiable images or data included in this article.

## Author contributions

VD-C, AA, DY, MC, DM, IA, VJ-J, JR, and EM performed experiments and analyzed data; J-LC, SB-D, JB, RR, TM, AT, and SB designed the project and supervised the work. VD-C, AA, MC, JB, and SB wrote and edited the manuscript. All authors contributed to the article and approved the submitted version.

## Funding

This work was supported by the Alfonso Martin Escudero Foundation (VD-C), Rosetrees Trust Foundation (VD-C, SB), and the Wellcome Trust (104807/Z/14/Z and 101788/Z/13/Z) (AT, RR, and DY). The project was sponsored by University College London (UCL) and the National Institute for Health Research Biomedical Research Centre at Great Ormond Street Hospital for Children NHS Foundation Trust. AA and EM were supported by the National Institute for Health Research UCLH Biomedical Research Centre. The Laboratory of Human Genetics of Infectious Diseases is supported in part by institutional grants from INSERM, Paris Cité University, Foundation for Medical Research (FRM) (EQU201903007798), St. Giles Foundation, The Rockefeller University Center for Clinical and Translational Science (8UL1TR001866), the National Center for Research Resources, the National Center for Advancing Sciences (NCATS), National Institutes of Health, the National Institute of Allergy and Infectious Diseases (5R01AI089970, 5R37AI095983 and U19AI111143) and grants from the French National Research Agency (ANR) under the “Investments for the future” program (ANR-10-IAHU-01) and GENMSMD (ANR-16-CE17-0005-01 for JB) grants. VJ-J was supported by LABEX IBEID. JR is supported by INSERM (“poste d’accueil”) and the MD-PhD program of the Imagine Institute with the support of the Bettancourt-Schueller Foundation.

## Acknowledgments

We thank the Alfonso Martin Escudero Foundation, Rosetrees Trust Foundation and the Wellcome Trust for their support. The content of the manuscript has previously appeared in the thesis: VD-C (2019) Investigating of the pathogenesis of JAK1 immunodeficiency. (dissertation/thesis). Madrid, Spain. Universidad Complutense (67).

## Conflict of interest

SB has received grant support from the European Union, National Institute of Health Research, UCLH and GOSH/ICH Biomedical Research Centers and CSL Behring and personal fees or travel expenses from Immunodeficiency Canada/IAACI, CSL Behring, Baxalta US Inc and Biotest. VD-C has received travel expenses from CSL Behring and Takeda. IH is employed by Healx. J-LC serves on the board for ADMA and Elixiron Immunotherapeutics and serves as a consultant for Celgene, Kymera TX, Pfizer, Nimbus, Sanofi, Vitae Pharmaceuticals Inc. and Asahi Kasei.

The remaining authors declare that the research was conducted in the absence of any commercial or financial relationships that could be construed as a potential conflict of interest.

## Publisher’s note

All claims expressed in this article are solely those of the authors and do not necessarily represent those of their affiliated organizations, or those of the publisher, the editors and the reviewers. Any product that may be evaluated in this article, or claim that may be made by its manufacturer, is not guaranteed or endorsed by the publisher.

## Supplementary material

The Supplementary Material for this can be found online at: https://www.frontiersin.org/articles/10.3389/fimmu.2022.888427/full#supplementary-material

## Glossary

**Table:**

BCG	Bacillus Calmette-Guérin
CFU	Colony forming units
CIITA	Class II Transactivator
CMV	Cytomegalovirus
DMEM	Dulbecco’s Modified Eagle’s Medium
DMSO	Dimethyl sulfoxide
DNA	Deoxyribonucleic acid
EBV	Epstein-Barr virus
EBV-B cells	Epstein-Barr Virus-Transformed B cells
FC	Flow cytometry
FCS	Fetal calf serum
GAPDH	Glyceraldehyde-3-phosphate dehydrogenase
*GFP*	*Green fluorescent protein*
GOF	Gain of function
HPV	Human papilloma virus
HSV	Herpes simplex virus
IFN-α	Interferon alpha
IFN-λ	Interferon lambda
IFN-γ	Interferon gamma
IFNAR	Interferon alpha receptor
IFNGR	Interferon gamma receptor
IL	Interleukin
IRF	Interferon regulatory factor
IU	International unit
JAK	Janus Associated Kinase
KD	Knock down
LOF	Loss-of-function
MFI	Mean fluorescence intensity
MHC	Major histocompatibility complex
MOI	Multiplicity of infection
MSMD	Mendelian susceptibility to mycobacterial disease
OD	Optical density
PBMCs	Peripheral blood mononuclear cells
PBS	Phosphate-buffered saline
PCR	Polymerase chain reaction
PFA	Paraformaldehyde
*PFU*	Plaque-forming unit
PI	Propidium Iodide
PID	Primary immunodeficiency
PIV5	Parainfluenza virus 5
PIV5VΔC	Attenuated recombinant strain of PIV5
PMA	Phorbol myristate acetate
P/S	Penicillin-streptomycin
qPCR	Quantitative real-time PCR
RNA	Ribonucleic acid
RT-PCR	Reverse transcription polymerase chain reaction
RT-qPCR	Real time-quantitative polymerase chain reaction
SC	scrambled control
SCID	Severe combined immunodeficiency
SDS-PAGE	Sodium dodecyl sulphate–polyacrylamide gel electrophoresis
SE	Standard deviation
SEM	Standard error of the mean
shRNA	Short hairpin RNA
STAT	Signal transducer and activator of transcription
TYK2	Tyrosine kinase 2
VSV	Vesicular stomatitis virus
VZV	Varicella zoster virus
WT	Wild type
γc	Common gamma chain
