# Supplementary material for: Partial human Janus kinase 1 deficiency predominantly impairs responses to interferon gamma and intracellular control of mycobacteria
Source: Front Immunol. 2022 Sep 9;13:888427. doi: 10.3389/fimmu.2022.888427 (PMC9501714; doi:10.3389/fimmu.2022.888427)
Supplement: Supplementary file 1 [file DataSheet_1.pdf]

Supplementary Methods

Culture of patient cells and human cell lines

THP-1 cells and Epstein-Barr Virus (EBV)-immortalized B cell lines (EBV-B cells) were maintained in RPMI-1640 medium containing 10%-20%heat-inactivated fetal calf serum (FCS) and 1% penicillin-streptomycin (P/S). Primary dermal fibroblasts from patients and healthy controlsand 293T cells were cultured in DMEM medium and 10% heat-inactivated FCS and 1% P/S. For the different experiments fibroblasts were detached using Accutase® solution (A6964, Sigma Aldrich). Regular checks for mycoplasma contamination were performed. JAK1-complented stable clones were kept in the presence of puromycin (3 ug/ml).

Lentivirus preparation and transductions

JAK1 knock down (KD) and scrambled control (Sc) cell lines were generated using lentiviral vectors expressing short hairpin RNA (shRNA) sequences.pGIPZ vectors carrying the short hairpin RNA against JAK1(TAGTACACACATTTCCATG) or scrambled control (TGAACTCATTTTTCTGCTC) sequences as well as puromycin resistance cassette and turbo-GFP marker for selection were supplied by University College London Open Biosystems (London UK). Lentivirus stocks were prepared by transfection of 293T cells (80-90% confluence) cultured in DMEM medium and 10% heat-inactivated fetal bovine serum, with the envelope plasmid 17.5 µg pMD.G2 (VSV-G/envelop), 32.5 µg p8.74 plasmid (gag-pol) and 25 µg vector construct with the transfection reagent PEI/Optimen following the manufacture instructions. Medium was replaced 5h post transfection and medium was harvested after 24 and 48 h, cleared by centrifugation (4,000 rpm, 5 min), filtered through 0.22 µm filters and left to spin for 2h 4ºC 50,000 g. Viruses were tittered on 293T cells by scoring GFP positive cells by flow cytometry 3 days post transduction. Virus stocks were stored at -80°C. Transductions of cells were carried out by infection at a multiplicity of

infection of 1:10 for 6h, and then the virus containing media was replaced by fresh media. Cells were selected in puromycin-containing medium (3 ug/ml) and the efficiency of transduction was assessed as percentage of GFP positive cells by flow cytometry. Lack of JAK1 expression in JAK1-deficient cells was verified by reverse transcription polymerase chain reaction *(*RT-PCR).

Western blot analysis

2x10^6 THP1 SC or THP1 KD cells were collected, washed with cold PBS, and lysed in RIPA (Sigma-Aldrich) lysis buffer containing 1X protease inhibitor cocktail (Roche) for 30min on ice. Cell lysates were centrifuged at 12000rpm for 20min at 4°C and supernatant was collected. Samples were subjected to SDS-polyacrylamide gel electrophoresis (10% Mini-Protean TGX Precast Protein Gels, Biorad) and transferred to nitrocellulose membranes (Biorad). After blocking with 5% BSA for 1h at room temperature, the membranes were probed with the following primary (overnight at 4°C) and HPR-conjugated secondary antibodies (1h at room temperature): mouse anti-JAK-1 (clone 73/JAK1, BD Biosciences), rabbit anti-GAPDH (clone D16H11, Cell Signalling Technology), anti-mouse-IgG-HRP (Cell Signalling Technology) and anti-rabbit-IgG-HPR (Cell Signalling Technology). The proteins were detected with SuperSignal West Pico Plus chemiluminescent substrate (ThemoFisherScientific) using the ChemiDoc Imaging System (Biorad).

Determination of mRNA levels by reversetranscription real time-quantitative polymerase chain reaction (RTqPCR) in THP1 cells

THP1 were left untreated or were treated with IFN-α 1000 IU/ml for 15h. Total RNA was extracted using Monarch Total RNA Miniprep Kit (New England Biolabs). RNA was reverse-transcribed using LunaScript RT SuperMix Kit (New England Biolabs) and used to quantify the expression levels of *MX1* and *OAS1* in triplicates. For quantification of cDNA,

qPCR was performed using CFX96 Touch Real-Time PCR Detection System (Biorad) with Taqman probes (ThermoFisher Scientific) labelled with FAM (Hs00242943_m1 for *OAS1*, Hs00895608_m1 for *MX1*, Hs02786624_g1 for *GAPDH*, and Hs02800695_m1 for *HPRT*). Fold changes were calculated using the ΔΔCT method and results were normalized to the levels of *HPRT* and *GAPDH*.

Mycobacterial and bacterial culture

The *Mycobacteriumbovis Calmette–Guérin* (BCG) Pasteur strains (ATCC® 35748™), BCG expressing-mCherry (kind gift from Prof. Brian Robertson Imperial College of London, London, U.K) and *Salmonella typhimurium* (ATCC 14028) (kind gift from Dr Dagmar Alber, UCL, London, U.K)were used in the study.

Mycobacteria were grown to mid-log phase (OD between 0.6-1) in Middlebrook 7H9 medium supplemented with 10% OADC enrichment medium (BD Biosciences), plus 50 μg/ml Hygromycin for BCG expressing-mCherry. Stock cultures were maintained in glycerol at –80° C until later use. Viable cell counts in thawed aliquots of BCG were determined by plating serial dilutions of cultures onto supplemented Middlebrook 7H11 agar plates followed by incubation at 37ºC for 14-21 days. Salmonella was grown to mid-log phase in LB broth overnight with agitation.

The MOI calculation was performed using the following conversion: OD of 1 = 1 x 10^8CFU/ml for BCG and OD of 1 = 10^9CFU/ml for *Salmonella*.Bacteria were washed and suspended in RPMI medium and 10% heat-inactivated FCS.

Microscopy

200,000 THP-1 cells were differentiated on 35mm glass bottom dishes (Fluorodish). After phagocytosis of BCG expressing-mCherry, cells were washed and incubated in complete medium in the presence or absence of IFN-γ (50 ng/ml) for 3 days. Subsequently, cells were incubated with 50 nMLysoTracker Deep Red (Life Technologies) for 30min, washed and

then fixed in 4% paraformaldehyde (PFA) for 10 min. Nuclei were stained with 5 µg/ml DAPI for 10 min, cells were then washed and kept on PBS. Cells were acquired using Leica inverted fluorescent microscope equipped with 60x oil objective for quantification of infected cells or Nikon Eclipse Ti-E confocal microscope equipped with 40x objectivefor colocalization analysis. Infected cells were counted manually and at least 100 cells per experiment were analysed. Images were processed using ImageJ (National institute of health) and Imaris image analysis software.

Viral assays

For the plaque assays, control and patient fibroblasts were grown in six-well dishes and infected with different dilutions of PIV5VΔC and PIV5 for 1h. Subsequently, 0.1% Avicel (FMC Biopolymer) was included in the overlay medium and cells were incubated for 5 days at 37ºC in 0.5% CO_2. Plaques were visualized by immunostaining using a pool of monoclonal virus-specific antibodies for the viruses as described previously (29,30), together with alkaline phosphatase-conjugated secondary antibody by using SIGMAFAST BCIP/NBT as the substrate.

Control and patient fibroblasts were grown on 13 mm diameter coverslips in individual wells of 24-well plates and then were left unstimulated or stimulated with different concentrations (10^3 - 10^4IU/ml) of IFN-α2b overnight. Cells were infected with PIV5 at a multiplicity of infection of 10 pfu/cell. The inoculum was adsorbed for 1 h and then cells were incubated in complete medium in the presence or absence of IFN for 24h. Monolayers were incubated in fixing solution (5% formaldehyde and 2% sucrose in PBS) for 15 min at room temperature, then permeabilized (0.5% Nonidet-P40 and 10% sucrose in PBS) for 5 min, and washed three times in PBS containing 1% FCS and 0.1% azide (PBS, 1% FCS, 0.1% azide). To detect the proteins of interest, cell monolayers were incubated with 10–15 µl of an antibody used to detect PIV5 as previously described (30). Cells were subsequently washed (PBS, 1%

FCS, 0.1% azide). In addition, cells were stained with the DNA-binding fluorochrome DAPI (0.5 μgml−1; Sigma-Aldrich) for nuclear staining. Following staining, monolayers were washed with PBS, mounted using Mowiol and examined using a Nikon Microphot-FXA immunofluorescence microscope.

EBV–B cells were either left untreated or were treated with 10^4 IU/ml IFN-α2b for 18 h. The kinetics of vesicular stomatitis virus (VSV) growth in EBV–B was determined by resuspending the cells in RPMI medium containing the virus inoculum and incubating for 1 h (VSV MOI = 1), washing with PBS and resuspending in fresh complete medium. Virus-containing supernatants were then collected at the indicated time points. VSV titers were determined by calculating the 50% end point (TCID50), as previously described (31), after the inoculation of 96-well plates with Vero cell cultures.

Supplementary Tables

Table S1.

Clinical diseases allelic with MSMD and viral susceptibility due to defects in type I and/or II IFN signalling pathways.

Disease	Mycobacterial	Viral	Other pathoges	Malignancy
JAK1 LOF AR	++	+/-		Bladder carcinoma
STAT1 LOF AR<br>-Complete<br><br>-Partial	 +++<br><br>++	 ++<br><br>+	 +	
STAT1 LOF AD	++			Liver carcinoma
IFNγR1 LOF AR<br>-Complete<br><br>-Partial	 +++<br><br>+	 ++<br><br>+	 +	B cell lymphoma<br>Pineal germinoma
IFNγR1 LOF AD	++			
IFNγR2 LOF AR<br>-Complete<br><br>-Partial	 +++<br><br>+			Cutaneous squamous carcinoma
IFNγR2 LOF AD	+			
TYK2 LOF AR	++	+	+	
STAT2 LOF AR		++<br>Disseminated vaccine strain measles		
IFNAR1 LOF AR		++<br>Severe disease caused by yellow fever, measles vaccine and COVID-19*		
IFNAR2 LOF AR		++<br>Disseminated vaccine-strain measles, HHV6		
IRF9 LOF AR		++<br>Severe influenza disease		

Adapted from Bustamante*et al.*Semin Immunol 2014 and Bousfiha*et al.* J Clin Immunol 2020. * Zhang Q *et al.*Science. 2020 Oct 23;370(6515) and Bastard *et al.* J Exp Med. 2021 Apr 5;218.

Table S2. Primers used for RT-qPCR and RT-PCR

Target	Forward (5' → 3')	Reverse (5' → 3')
JAK1	TGGATCTCTTCATGCACCGGA	ATGAATGGGCCACACTCACTG
IRF1	CAGAGAAAAGAAAGAAAGT	CATCAGAGAAGGTATCAG
CIITA	ATG CGC TGA GTG AGA ACA AGA TC	GGAAGCGGAGGTGAGGAGATT
GAPDH	GAGCCACATCGCTCAGACAC	CATGTAGTTGAGGTCAATGAA GG
β-Actin	CAGCAAGCAGGAGTATGACG	AAAGCCATGCCAATCTCATC

RT-qPCR= Real timequantitative polymerase chain reaction, RT-PCR= Reverse transcriptionpolymerase chain reaction, JAK1= Janus associated kinase 1, IRF1= Interferon regulatory factor 1, GAPDH= glyceraldehyde-3-phosphate dehydrogenase

Supplemental Figures

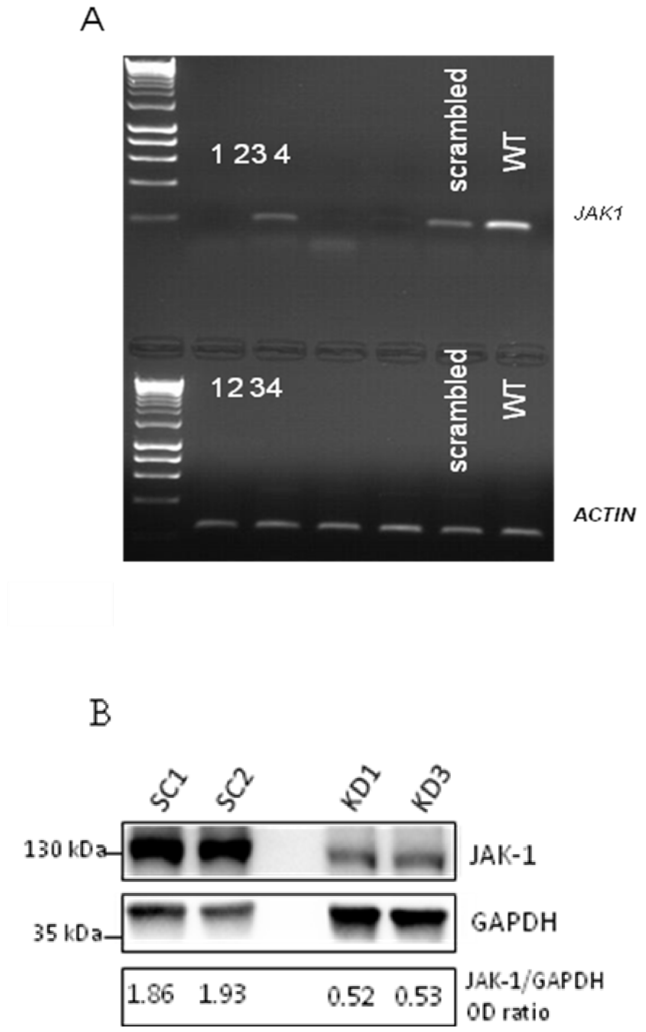

Figure S1.

JAK1 expression in JAK1-deficient cell lines and controls.

THP1 cells were transfected with vectors expressing JAK1 shRNA and scrambled control shRNA. (A) RT-PCR analysis of JAK1 expression is shown. Data is representative of two independent experiments. (B) Lysates for western blot were prepared from $2x10^6$ THP1 SC or KD cells. Membranes were incubated with mouse anti-JAK-1 (clone 73/JAK1, BD Biosciences) and rabbit anti-GAPDH (Cell Signalling Technology) followed by anti-mouse-HRP and anti-rabbit-HRP (both from Cell Signalling Technology), respectively.

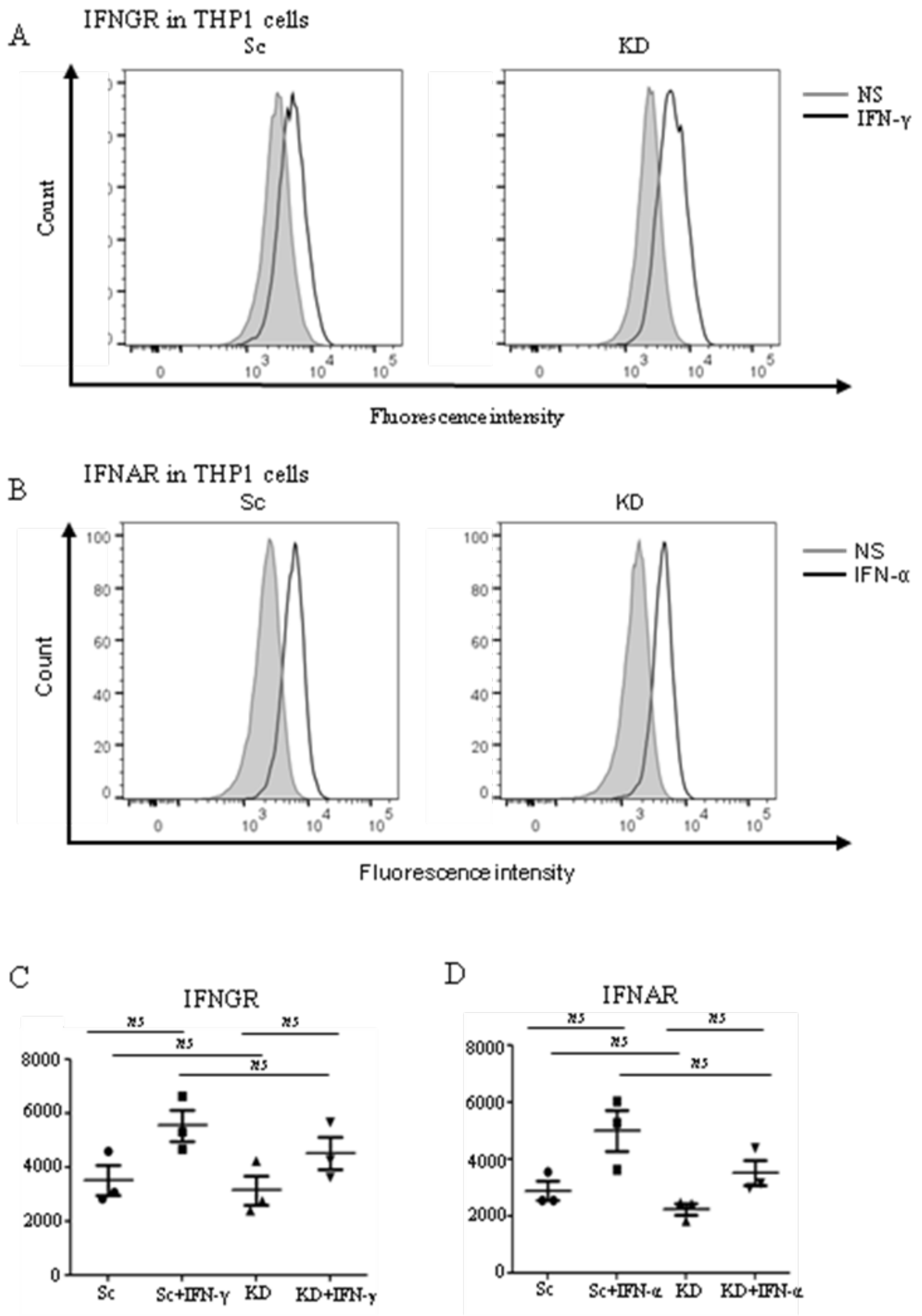

Figure S2.

Analysis of IFNGR and IFNAR expression in KD and Sc THP1 cells by flow cytometry.

(A,C) IFNGR and (B,D) IFNAR expression in THP1 cells after stimulation with or without IFN-γ and IFN-α respectively. Data are from three independent experiments. One-way ANOVA with Tukey's Multiple Comparisons Test. *P <0.05.

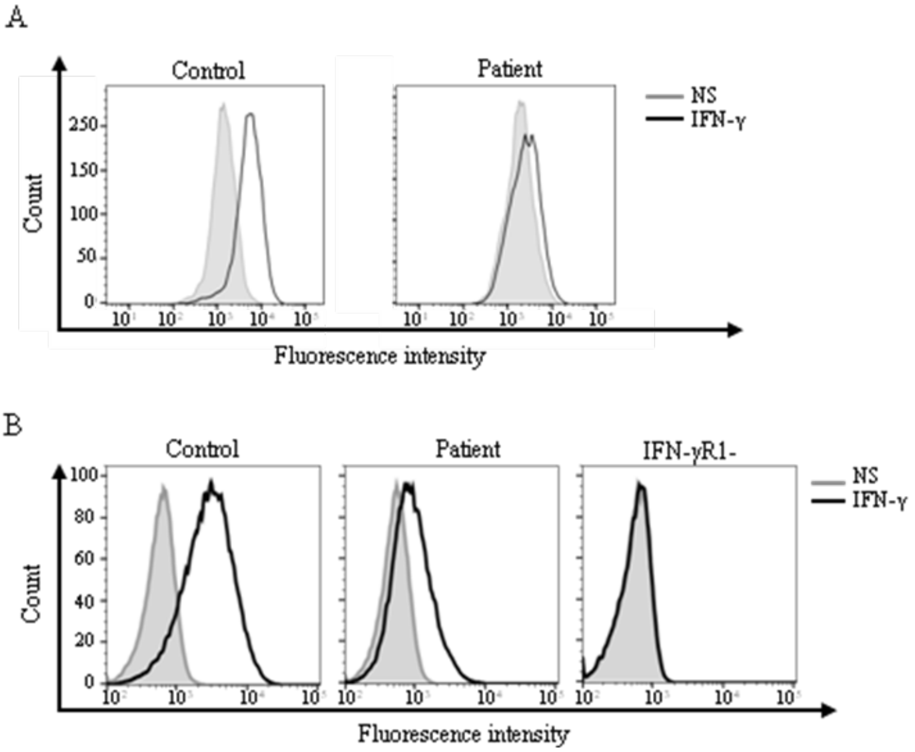

Figure S3.

Analysis of pSTAT1 expression in fibroblasts and EBV B-cells by flow cytometry.

(A-B) Analysis of JAK/STAT signaling by FC in control and patient fibroblasts and EBV-B cells after stimulation with IFN-γ. A and B display a representative experiment.

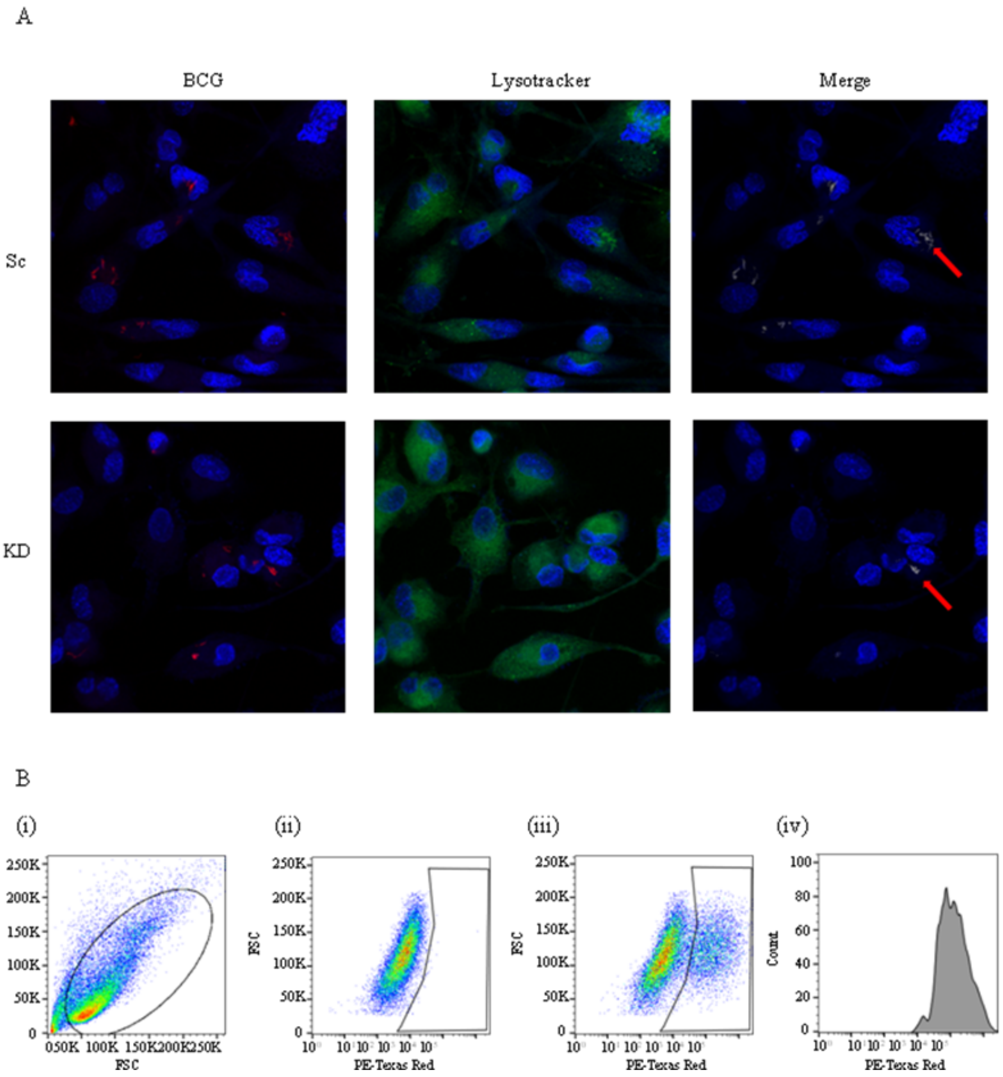

Figure S4.

BCG is internalized and localised into acidified compartments in control and JAK1-deficient THP1 cells.

(A) Internalization of mCherry-BCG by macrophages differentiated from the Sc and KD THP1 cell lines, unstimulated or stimulated with IFN-γ. Acidified phagosomes containing mCherry-BCG are shown with an arrow. (B) Gating strategy for analysis of phagosome acidification by flow cytometry: (i) SSC vs FCS density plot: a gate has been applied to identify the THP1 cell population excluding debris. (right) (ii) Overlay of a negative unstained population allows easy identification of the positive cells. (iii,iv) Cells within the gate defined are represented in a histogram to evaluate the relative fluorescence.

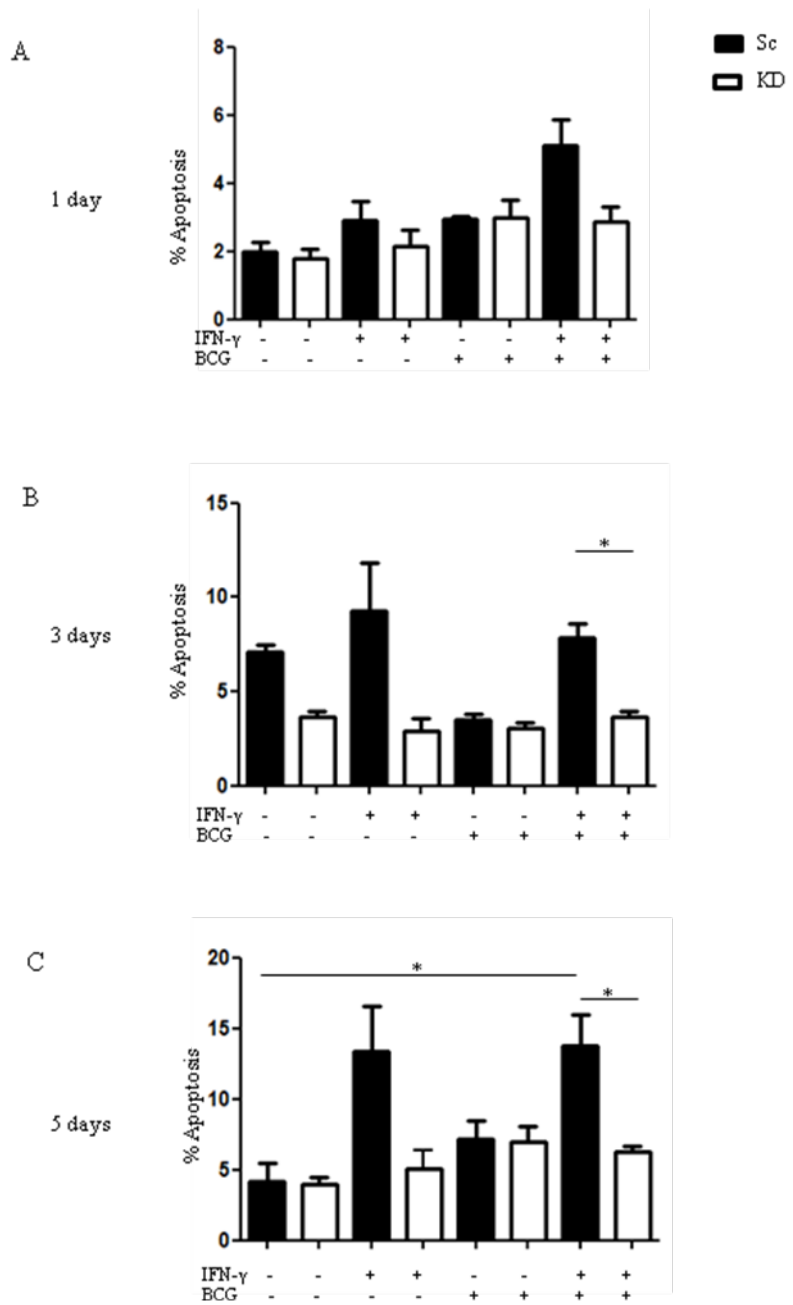

Figure S5.

Apoptosis is reduced in JAK1-deficient THP1 cells.

(A,B,C) Percentage of apoptosis quantified by flow cytometry using annexin V / PI staining in THP1 cell lines at different time points following BCG infection, with or without prior IFN-γ stimulation. Data are from four independent experiments. Two-tailed Mann Whitney test. *P <0.05.

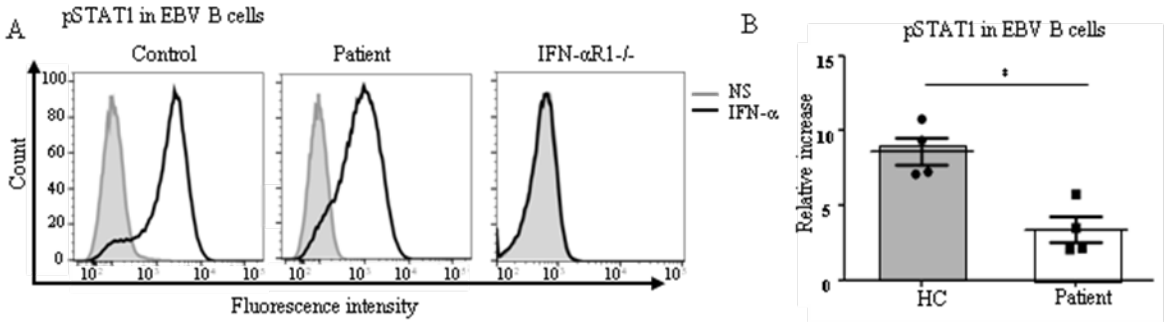

Figure S6.

Analysis of pSTAT1 expression in EBV B-cells by flow cytometry.

(A-B) Analysis of JAK/STAT signaling by FC in control and patient EBV-B cells after stimulation withIFN-α. Data are from four independent experiments. Two-tailed Mann Whitney test. *P <0.05.

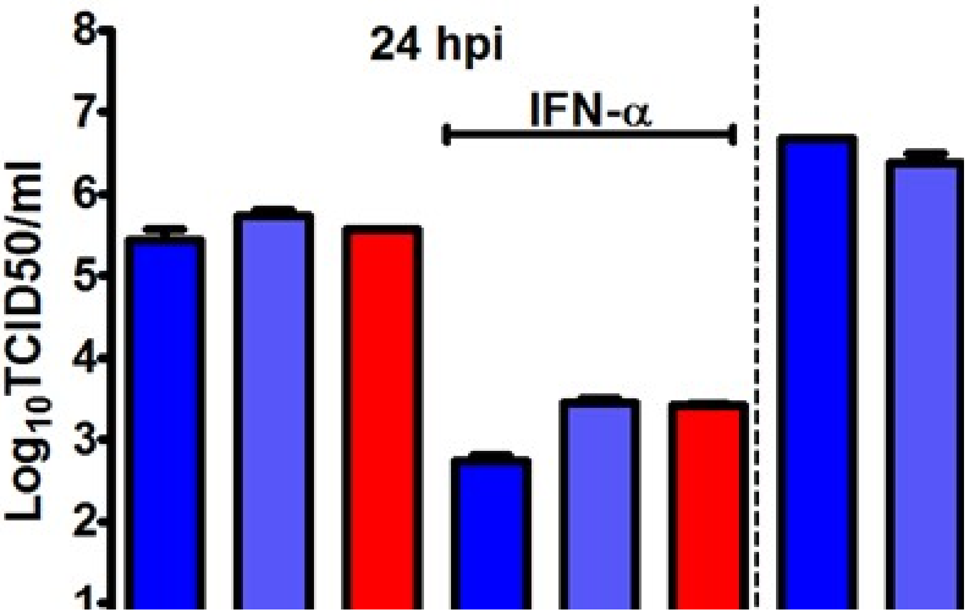

Figure S7.

HSV-1 Viral assay.

Control and patient fibroblasts were left untreated or were treated with 105 IU/ml IFN-α for 24 hours. They were then infected by incubation with various HSV-1 (moi=0.1), and after 48 hours, viral titers were determined by visualizing cell lysis.
